# Supplementary material for: The prognostic value of thromboelastography MA/R ratio in predicting mortality in acute respiratory failure patients
Source: PLoS One. 2026 Feb 5;21(2):e0340360. doi: 10.1371/journal.pone.0340360 (PMC12875460; doi:10.1371/journal.pone.0340360)
Supplement: S1 Table — (DOCX) [file pone.0340360.s001.docx]

| **S1 Table. Thromboelastography (TEG) parameters: definitions, clinical significance, and reference ranges.** | | | | | |
| --- | --- | --- | --- | --- | --- |
| TEG Parameters | Definition | | | Significance | Reference |
| R (min) | The time from the start of the assay until initial fibrin formation. | | | Indicates the time it takes for clot initiation. Prolonged R time suggests coagulation factor deficiency or anticoagulant effects, while a shortened R time indicates a hypercoagulable state. | 5.0 - 10.0 |
|  |  | | |  |  |
| K (min) | The time from the end of R until the clot reaches a fixed strength. | | | Reflects the speed of clot formation. Prolonged K time can indicate deficiencies in fibrinogen or platelet function. | 1.0 - 3.0 |
|  |  | | |  |  |
| α-angle (deg) | The angle formed by the slope of the TEG curve following the R time | | | Represents the rate of clot strengthening. A lower α angle suggests impaired fibrin formation or platelet function, while a higher angle indicates rapid clot formation. | 53.0 - 72.0 |
|  |  | | |  |  |
| MA (mm) | The maximum amplitude achieved by the TEG tracing. | | | Reflects the overall strength of the clot, primarily influenced by platelet function and fibrin interaction. A low MA suggests platelet dysfunction or low fibrinogen levels, while a high MA indicates hypercoagulability. | 50.0 - 70.0 |
|  |  | | |  |  |
| MA/R ratio  (mm/min) |  | MA |  | The MA/R ratio provides insight into the overall coagulation balance, reflecting both clot strength (MA) and the efficiency of clot formation (R time). A high MA/R ratio typically suggests a hypercoagulable state, where clot strength is disproportionately high relative to the time needed to initiate clotting. Conversely, a low MA/R ratio can indicate hypocoagulability or a coagulation factor deficiency, where clot strength is reduced despite normal or prolonged clot initiation times. | / |
|  |  | R |  |  |  |
